# Public confidence in the police: using opinion survey data to explore the current ‘moment’ in British policing

**DOI:** 10.1080/10439463.2025.2508198

**Published:** 2025-06-03

**Authors:** Ben Bradford, Christine Weirich, David Rowlands, Adam Crawford

**Affiliations:** a Department of Security and Crime Science, University College London, London, UK; b ESRC Vulnerability and Policing Futures Research Centre, University of Leeds, Leeds, UK; c ESRC Vulnerability and Policing Futures Research Centre, University of York, York, UK

**Keywords:** Trust, legitimacy, police, public opinion

## Abstract

On many accounts British policing is currently experiencing a legitimacy crisis, a claim frequently evidenced by data from opinion surveys. Such surveys have been in the field for more than half a century, generating a wealth of data demonstrating the waxing and waning of ‘trust and confidence’ in police. Yet, few nationally representative surveys field items that cover the full range of public attitudes towards police. In this paper, we present results from a representative survey of England, Scotland and Wales (*n* = 1,484) that fielded items tapping into a wide range of attitudes towards police, including trust, legitimacy, and measures of confidence developed through a novel series of deliberative focus groups that sought to define a ‘minimum policing standard’. We show that few people feel police are meeting these standards, and that legitimacy does indeed seem low. Views of policing are currently marked by high levels of uncertainty, disappointment, and disillusion. However, while *confidence* and *legitimacy* are low, public *trust* seems to be higher. Moreover, different aspects of police performance – or at least people’s judgements of it – have different associations with overall confidence, trust and legitimacy. Visibility and ‘presence’ seem more important for overall confidence, while fairness and proportionality are more important for legitimacy. Our findings both offer support to the idea police-public relations are under significant strain and provide insight into why this is so.

## Introduction

Surveys of public opinion of the police have a long history in the UK. The 1962 Royal Commission on the Police funded a national survey probing public views of the police. In the 1970s, Sparks *et al.* (1977) surveyed Londoners on their experiences of crime and attitudes towards the criminal justice system, while in the early 1980s the Policy Studies Institute project ‘Police and People in London’ had a tighter focus on policing (Smith 1983). From 1982 onwards the British Crime Survey (now the Crime Survey of England and Wales – CSEW) fielded items on attitudes towards the police, courts and other elements of the criminal justice system (Mayhew and Hough 1983). The ‘Public Attitudes Survey’ conducted by the Mayor’s Office for Policing and Crime in London has been running for nearly two decades and provides unparalleled detail on trust in the police and related issues in the capital (Dawson *et al.* 2023). Police organisations and Police and Crime Commissioners throughout the country regularly conduct surveys covering their jurisdiction or purview. More widely, surveys of attitudes towards the police are now common on a global scale, with local, national, and international studies either focussing specifically on this issue or including at least some relevant survey items (e.g. Cao *et al.* 2012, Kääriäinen and Sirén 2012, Kaasa and Andriani 2022).

Through such efforts, we now know a huge amount about what people think about police. In the UK, the focus of the current paper, trend analysis of the CSEW and other sources has documented, for example, a decline in confidence in the police from the 1980s to mid 2000s, then an increase to about 2017 and, recently, a marked decline (Bradford 2024). Survey data have also been used to try to better understand such shifts in public opinion. A plethora of studies have considered the factors that shape public trust and confidence, legitimacy, and a range of associated constructs (for reviews see Brown and Reed Benedict 2002, Walters and Bolger 2019, Bolger *et al.* 2021). The recent, and significant, decline in public confidence has been linked to: scandals rocking the police, notably but not only in the Metropolitan Police; the longer-term effects of austerity cuts to police budgets, specifically neighbourhood policing, affecting visibility and engagement with communities; and the wider social and economic effects of austerity, Brexit, and the pandemic (Bradford 2024).

Talk of a crisis of trust, confidence and even legitimacy is currently commonplace (Bradford *et al.* 2024), triggering significant policy attention and other activity (e.g. Brown and Hobbs 2023, MPS n.d.). Results from surveys feed into, inform and react to policy development and police practice (Stanko and Dawson 2016). With the decline in public confidence, such efforts have moved once again into research and policy agendas (Kimaram *et al.* 2023, Pickering *et al.* 2024), and the new Labour government’s recently announced Community Policing Guarantee appears to recognise impacts of reduced police visibility on public perceptions and the role neighbourhood policing can play in fostering trust and confidence.

Despite the level and reach of this activity, though, few surveys with nationally representative samples have fielded suites of questions that address the nuances of public opinion, or indeed the full range of concepts – satisfaction, trust, confidence, legitimacy – that are used to describe police-public relations. In this paper, we present results from a representative survey of England, Scotland and Wales that fielded a large set of items covering these different concepts. We introduce a new set of measures of public confidence in the police, developed via an iterative focus group-based approach that explored attitudes towards local – and ‘low’ (Brodeur 2010) – policing, and we add to these measures of trust and legitimacy. Our analysis illustrates the relationships between these associated, yet conceptually distinct, aspects of public opinion, and casts light on the recent decline in police-public relations.

The paper proceeds as follows. First, we provide an overview of the measurement of public attitudes towards the police, before going on to outline the new survey items developed for the current study. After a section on data and methods, we present descriptive statistics and then multivariate analysis. We conclude with consideration of what the findings from the survey tell us about the current state of police-public relations, how we got to where we are, and what might be done about it.

## Surveying public attitudes towards police

Studies of public attitudes towards police have tended to be interested in one or more of a number of key constructs. These have included, first, ‘satisfaction’, which usually refers to a retrospective judgement about specific encounters with officers or services police have delivered (Cao 2015): active, conscious, assessments of a particular experience of policing.

Second, especially in the UK research has been concerned with the notion of ‘trust and confidence’, which has been used as a kind of umbrella term to cover general attitudes towards police (Jackson and Bradford 2010; Morell *et al*. 2018). Many researchers would suggest, though, that trust and confidence are two slightly different things. Trust refers to a willingness to be vulnerable to another under conditions of risk, where such willingness is based on positive evaluations and expectations of the other’s competence, benevolence and good intentions (Mayer *et al.* 1995, Hamm *et al.* 2017). Confidence, by contrast, refers to a ‘conscious evaluation’ (Cao 2015, p. 242) of whether an entity is trustworthy (i.e. whether it is in fact competent, benevolent and well-intentioned). One implication of this distinction is that while we all have some level of *confidence* in police – or at least will formulate one when prompted to do so – only on some occasions will some of us need to actually *trust* police, for example by calling them to report a crime and thus involving them in our lives. These are fine distinctions, though. One may evince trust in the police in a more abstract manner that does not imply direct action, for example by accepting that valued outcomes, such as security and public order, are placed in their hands (Hamm *et al.* 2017).

Third, influenced by procedural justice theory (Tyler 2006a), a wide range of studies have fielded measures of procedural justice (which refers to the quality of police decision-making and treatment, and specifically issues of voice, respect, neutrality and trustworthy motives; Jackson and Gau 2016) and legitimacy. There continues to be a debate about what legitimacy ‘is’, and how it should be measured (Cao and Graham 2019, Jackson and Bradford 2019, Trinkner 2019), but most studies have used some combination of subjective ‘duty to obey’ police (Tyler 2006b), institutional trust in police, and ‘normative alignment’, the extent to which people believe the values of police are appropriate and aligned with their own (Tyler and Jackson 2014). Fourth, the outcomes of trust and legitimacy have also been an area of interest, perhaps most frequently represented by items gauging people’s readiness to cooperate with police in some way (Bolger and Walters 2019).

As well as being conceptually similar, the constructs described above are linked to one another in potentially causal chains. Judgements about officers’ behaviours during interactions, which might be based on perceptions of procedural justice and lead to satisfaction or dissatisfaction, feed into confidence, trust and legitimacy, for example (Mazerolle *et al.* 2013, Langley *et al.* 2021). Legitimacy is premised in confidence and perceptions of trustworthiness – people judge the normative appropriateness of policing in light of their assessment of whether police are behaving in the ‘right’ way by being competent, well-intentioned, and benevolent (Hamm *et al.* 2017, Jackson 2018). And, as noted, cooperation is thought to flow from trust and legitimacy.

### Features of the current evidence base

All five constructs outlined above have been extensively covered in small-scale and local surveys, and lab and field experiments, which have developed a sophisticated understanding of the formation, reproduction and implications of police-public relationships. However, the focus of the large-scale population surveys of the kind frequently cited in public and policy debates has most often been on confidence or perceptions of trustworthiness. Surveys field items that relate to aspects of police performance and behaviour which are implicitly or explicitly assumed to be important for, or indicators of, confidence and/or trust. The CSEW, for example, included in its 2019/20 sweep items such as (do you agree or disagree that …): (i) ‘They (the police in this area) can be relied on to be there when you need them’; and (ii) ‘They (the police in this area) would treat you with respect if you had contact with them for any reason’ (Kantar Public/Office for National Statistics n.d.). Other items are more summative in nature. A key CSEW item, fielded over many years and across multiple different surveys, is ‘Taking everything into account, how good a job do you think the police IN THIS AREA are doing?’, with response categories of excellent, good, fair, poor and very poor. This is usually interpreted as a measure of ‘overall trust and confidence’. By contrast, international surveys, such as the ESS, unless specifically focussed on police tend to include items directly probing subjective trust, such as ‘How much do you trust the police?’ (0 no trust at all – 10 complete trust) (ESS 2023).

The survey evidence base pertaining to public attitudes towards the police – certainly in the UK and often further afield – also has several other features that, arguably, inhibit nuanced understanding of the phenomena at hand. First, there is significant overlap in the ways the central constructs have been measured. In their systematic review of satisfaction with police, Bolger *et al.* (2021, p. 4) define their dependent variable as ‘citizen global perceptions of satisfaction with the police’, but go on to note that this concept:

currently lacks sufficient precision in both conceptualization and operationalization … studies revealed a wide variety of definitions from general satisfaction, perceptions of confidence (mostly regarding perceptions of confidence in crime-control), perceptions of trust, and a mixture of all three. Many studies also failed to provide any conceptual definition of the dependent variable.

Others have made similar assertions about the measurement of satisfaction, trust, confidence and/or legitimacy, and indeed procedural justice, which, while clearly referring to different aspects of peoples’ experiences of police, have been measured by a wide range of different but frequently over-lapping survey items (e.g. Jackson and Gau 2016; Yesberg and Bradford 2021). This is an issue for several reasons, not least because of the putative causal chain linking satisfaction (with a particular experience of policing) to confidence and/or perceptions of trustworthiness, and on to trust and legitimacy. If these concepts are measured by similar, over-lapping, items, this undermines our ability to properly specify the relationships between them.

Second, many surveys field the same questions over time and over different contexts for comparative reasons. Items first fielded in the CSEW, for example, are often used in other UK surveys, partly because this allows them to be benchmarked against the CSEW itself. This is an important feature of survey design, since it allows for standardisation, replication, and the creation of consistent time series. It is precisely this research that has enabled us to identify the recent decline in police-public relations. But because it relies on fielding established questions, this approach may have something of a blind-spot in determining the issues people really care about; something that might be particularly important at the present point in time, when people’s views on a whole range of societal institutions appear to be increasingly politicised, in flux and, often, declining (The Policy Institute 2023).

Third, nationally representative estimates of some of the central concepts in current debates in and around policing – most notably legitimacy – remain rare. Because many surveys concentrate on confidence and trustworthiness, and/or rely on single item measures, we know less than we perhaps should about how, at the population level, all these concepts relate to one another. Is it the case, for example, that a decline in confidence in police can be interpreted as a decline in legitimacy, as much current commentary assumes (e.g. Jacques 2023)? Or do police draw on other sources of legitimacy that might counteract this effect (see Factor and Mehozay 2023)?

In the current study, we attempt to address some of these lacunae. Assuming that confidence and trustworthiness are foundational to people’s attitudes towards the police, we conceptualise and operationalise these constructs on a ‘bottom up’ basis, deriving a set of questions gauging confidence in police based on what people told us was relevant to them. To do this, we used a focus group methodology to explore views of the service delivered by police, and from this constructed a set of survey indicators that we fielded in a nationally representative online survey. Asking respondents in a survey context whether they believed the police ‘delivered’ on what was important allows us to make comparisons between confidence, trust and legitimacy, trace the associations between them, and derive a clearer sense of the ‘crisis’ currently facing policing.

## The minimum policing standard

To generate an understanding of what people think is important in policing – and therefore criteria against which they judge whether police are trustworthy – we drew methodological inspiration from the on-going development and analysis of the ‘Minimum Income Standard’ (MIS) by a team from Loughborough University^1^ and the Joseph Rowntree Foundation. The Minimum Income Standard (MIS) ‘presents a vision of the living standards that we, as a society, consider everyone in the UK should be able to achieve’ (Padley and Stone 2023, p. 1). It uses an iterative focus group methodology to derive an understanding of what people think are the ‘basics’ of a good life in the UK at the current point in time – the set of material and other amenities everyone should have access to, and therefore be able to afford (Davis *et al.* 2015).

We describe in detail elsewhere the process through which we developed the Minimum Policing Standard (MiPoS) by drawing upon this prior work (Bradford *et al.* forthcoming). Briefly, we concentrated on the first part of the MIS process to develop a set of behaviours, actions and outcomes people thought the police simply should, under normal circumstances, be able to achieve. We limited discussion to ‘neighbourhood policing’, broadly defined: to the types and forms of policing that take place in and are tied to people’s neighbourhoods and their everyday lives. This includes the policing of crimes such as domestic violence, on the one hand, and online fraud, on the other: both happen to ‘ordinary’ people in ‘ordinary’ places. But ‘high’ policing directed towards terrorism and other state-level threats was out of scope, as was the police response to serious crimes such as murder and rape, both because people tend to be very unfamiliar with these crimes and because we expected near 100% agreement that police should have the capability to address them. This emphasis on local or neighbourhood policing mirrors the content of many UK-based and other surveys, which often concentrate on the forms of policing most familiar to the public.

Over three rounds of focus groups held in four different parts of the country, we asked a total of 93 participants to generate, refine and validate a set of minimum standards they expected – or at least hoped – police in their area would be able to meet. The final set of criteria are shown in Table 1. Participants identified three domains, which they labelled: (1) *Response*, (2) *Behaviour and Treatment*, and (3) *Presence and Engagement*. Response refers, broadly, to how the police should respond to and deal with calls for assistance, Behaviour/Treatment to how the police should behave while interacting with members of the public, and Presence/Engagement to requirements for the visibility and availability of police within neighbourhoods.

**Table 1.** The minimum policing standard.

Police Service Domains		
Response	Behaviour/Treatment	Presence/Engagement
Fast and proportionate response	Building trust	Greater community police presence (including on foot)
Focus on public safety	Treating the public with fairness and respect	Ability to speak directly to a person about local problems
Investigating and solving crimes	Building relationships within the community	Adequate follow-up in the aftermath of crimes
Openness and honesty when dealing with the pubic	Behaving in a professional manner	Responsive to the local community
Following up on crimes	Being role models of good behaviour	Physical local police station
Crime prevention and early intervention	Establish relationships with young people	Local community Police officer
Equal service across groups and places		Engaging in non-traditional types of communication with community

From these ‘minimum standards’ we developed a set of 18 survey indicators, shown in Table 2. This was itself an iterative and interpretative process. It would not have been possible to simply transpose the statements developed in the focus groups into a survey questionnaire. Rather, we took the ideas expressed in Table 1 and converted them into survey items that we hoped would be easily understood by people who had not engaged in the discursive focus group process. Most of the resulting items were worded in such a way as to allow responses on agree/disagree scales. While less than ideal from a methodological standpoint (Saris *et al.* 2010), we took the view that this made answering them easier than if response categories had been framed in a way truer to the idea of the MiPoS, which would seem to suggest more definite, indeed possibly binary, yes/no responses. It can be very hard for people to form such a view of police activity, not least because many will be unfamiliar with that activity. The items fielded in the survey are thus best interpreted as measures of confidence or perceptions of trustworthiness (we use these terms interchangeably). They gauge respondents’ assessments that police can be trusted to deliver across the various criteria set out by the focus group work.

One noteworthy feature of the MiPoS relates to what might be termed the ‘holistic’ way participants thought about policing. It is common in studies of public opinion to distinguish between instrumental and relational (and/or affective) aspects of peoples’ judgements on, attitudes about and orientations towards police (e.g. Sunshine and Tyler 2003, Sun *et al.* 2014, Bradford *et al.* 2016, Na *et al.* 2023). On these accounts, people draw a distinction between questions of (a) effectiveness and efficiency and (b) fairness and the relationship between police and public. Most studies then go on to show that people seem to value and attend to the relational aspects of policing far more than the instrumental. For example, perceptions of procedural justice are frequently found to be stronger predictors of outcomes such as trust, legitimacy and cooperation than perceptions of effectiveness.

The distinction between instrumental and relational factors is useful on theoretical and policy-related grounds. It offers insights into the social and psychological processes that underpin trust, confidence and legitimacy, and it offers evidence for critiques of policing styles that over-emphasise ‘toughness’ and ‘crime-fighting’ at the expense of fairness and relationship building (Tyler and Nobo 2022). Yet, when we start our analysis from a consideration of the ways people actually think and talk about policing, we find the difference between the instrumental and relational, effectiveness and fairness, largely if not entirely falls away. Participants in the focus groups placed both instrumental (e.g. *Make an effort to investigate crimes reported to them*) and affective (e.g. *Are open and transparent about the decisions they make*) concerns in the ‘Response’ domain, for example; and, overall, the MiPoS domains each cover instrumental *and* affective factors.

## The current research

In this paper we seek to add to current understanding of public attitudes to the police in the UK by presenting results from a nationally representative survey of England, Scotland and Wales that included measures of confidence, trust and legitimacy that both complement and extend existing survey question sets. We present evidence not only on ‘levels’ of public opinion, but on how different aspects of confidence in police (or perceptions of the trustworthiness *of* police), as defined in the MiPoS, are associated with overall levels of confidence, trust, and legitimacy. Four research questions motivate our analysis.

RQ1. Do British residents believe that their local police meet the criteria laid out in the MiPoS?

RQ2. How do levels of confidence in police, as represented by the MiPoS items, compare with other perceptions of and judgements about police?

RQ3. What is the strength of the association between confidence in the standard of police services and overall confidence in police, trust, and legitimacy?

RQ4. What do answers to these questions tell us about the current state of police/public relations?

## Data and methods

The data used in this study are drawn from the Public Voice panel managed by Verian (formerly Kantar Public), who were commissioned in the autumn of 2023 to run a survey with a target respondent sample of 1,500. The target population was GB individuals aged 18+ and living in residential accommodation. The sample was split: 1,000 GB-wide, plus a ‘boost’ of 500 from among those living in the most deprived fifth of each country (England, Scotland and Wales).

At the time the survey was conducted (November 2023), the Public Voice panel comprised 22,142 members in England, Scotland and Wales. Most were recruited via the ‘ABOS’ method in which (probabilistically) sampled individuals complete a 20-minute recruitment questionnaire either by web or on paper.^2^ Recruitment surveys were carried out in 2019, 2020 and 2021 and the respondent samples have been linked together via a weighting protocol to form a single panel. The sample for the MiPoS survey was drawn from among these 22,142 members. The panel was stratified by the Neighbourhood Index of Multiple Deprivation, and then by sex/age, before a systematic random sample was drawn. In total, 4,888 panel members were issued to the field, and the survey was closed on 20/12/23 with 1,517 completes, of whom 1,484 failed quality control tests and constitute the basic sample used in this paper. The overall conversion rate was 30%. All surveys were completed online, and those who completed the survey were offered a £10 voucher.

### Key measures and constructs

The final MiPoS items fielded in the survey are shown in Table 2. To reiterate, while these were all derived from the criteria developed in the focus group work changes were made to clarify and simplify for the survey context. For example, we dropped ‘engaging in non-traditional types of communication’ on the basis that someone coming ‘cold’ to this question might fail to grasp its meaning or relevance. We also sorted items so that similar issues or concepts did not appear in more than one domain. ‘Adequate follow up on crimes’ was dropped from Presence/Engagement, for example, on the basis that it was too similar to ‘follow up on crimes’ in the Response domain. Response categories for the Response and Behaviour/Treatment items were on a 5-point scale (Strongly agree; agree; neither/nor’; disagree; strongly disagree), while for the Presence/ Engagement items responses were on a 4-point scale (All of the time; most of the time; not very often, never). Don’t know responses were allowed.

We also included measures of overall confidence, trust, and legitimacy. Again, we used both the individual items and scales derived from these items (see Table 3 for full item wordings). *Overall confidence in the police* was measured by a single indicator taken from the CSEW: ‘Taking everything into account, how good a job do you think police do in this area’ (5-point scale: excellent, good, fair, poor, very poor). *Trust in the police* was measured by three items derived from Hamm *et al.* (2017) (5-point agree/disagree scale, as above). In line with prior studies (see Jackson *et al.* 2023) we treat police legitimacy as a two-component construct. Using items derived from Posch *et al.* (2021), three items measured *normative alignment with police* (5-point agree/disagree scale, as above), and three *duty to obey* police (7-point anchored scale, 0 = not at all my duty; 6 = Completely my duty). Don’t know responses were again allowed.

### Analytic strategy

To address our research questions analysis proceeds as follows. In relation to RQ1 and RQ2, we present univariate statistics on the views of people on the MiPoS items, as well as measures of overall confidence, trust and legitimacy.

To assess the association between confidence in police as represented by the MiPoS and overall confidence, trust, and legitimacy – RQ3 – we considered first the scaling properties of the MiPoS items. We used Confirmatory Factor Analysis (CFA) in the statistical package Mplus 7.2 to assess whether they can be used to construct attitudinal scales representing confidence across the three domains specified by the focus groups. Second, we used the resulting scales as independent variables in a series of regression models predicting each of the other constructs of interest: overall confidence, trust, normative alignment and duty to obey (we again used CFA to derive and validate scales of trust and legitimacy, using the relevant items shown in Table 3). Included as covariates in these models were measures of satisfaction with police contact, initiated by the individual or initiated by police, that occurred in the last 12 months (as two sets of three dummy variables representing satisfactory, unsatisfactory and ‘neutral’ (neither/nor) contact, with the reference category no contact). We also included as controls a set of variables regularly used as predictors of opinions of police (see *inter alia* Brown and Reed Benedict 2002, Bolger *et al.* 2021, Lim and Kwak 2022): age, gender, ethnicity and disability status; perceptions of disorder^3^; and victimisation and worry about crime.^4^ To these we added measures intended to capture something of the current economic and social atmosphere in the UK: two indicators of economic status, economic precarity (represented by the sum of two items that asked respondents how well they were managing on their present income and whether they would be able to borrow money if they needed it) and the IMD decile of their LSOA of residence;^5^ and two measures of political affiliation and engagement: vote at last election and newspaper readership.

## Results

Table 2 provides nationally representative estimates for assessments across the MiPoS domains. In line with other recent polling (e.g. YouGov n.d.), these make sobering reading for police. Between a fifth and a third of people agreed that police met each of the criteria within the Response domain (in all cases the largest group chose the indeterminate ‘neither/nor’ response). Responses in the Behaviour/Treatment domain were rather more positive, with between a third and nearly two thirds of people agreeing that police met the various criteria. Recall that the items in the Presence/Engagement domain used different response categories (4-point scale, all the time to never); here responses were also largely negative (and only around 5% of people thought police met all three criteria ‘all the time’).

**Table 2.** Minimum policing standard domains.

Percentages				
	Agree or strongly agree	Neither agree nor disagree	Disagree or strongly disagree	Don't know
**Response Domain**				
Prioritise public safety when deciding how to act	*36*	*32*	*9*	*23*
Provide a fast response	*25*	*29*	*26*	*19*
Prioritise the crimes most affecting your community	*22*	*32*	*20*	*25*
Provide responses that are proportionate to the issues involved	*28*	*32*	*16*	*24*
Deal with everyone in the same way, regardless of who they are	*33*	*24*	*22*	*21*
Make an effort to investigate crimes reported to them	*29*	*26*	*26*	*19*
Are open and transparent about the decisions they make	*23*	*33*	*20*	*24*
Provide adequate follow ups after a crime has been reported	*20*	*29*	*24*	*27*
Deal effectively with violent crimes	*30*	*29*	*14*	*28*
**Behaviour and Treatment Domain**				
Behave in a professional manner	*62*	*17*	*5*	*16*
Act in ways that build trust	*45*	*28*	*11*	*16*
Build relationships with the community	*34*	*31*	*17*	*18*
Treat people with respect	*51*	*26*	*8*	*15*
Have good relationships with young people	*23*	*31*	*17*	*30*
Provide role models of good behaviour	*37*	*32*	*13*	*17*
		All or most of the time	Not very often or never	Don't know
**Presence and Engagement Domain**				
Responsive to the local community		*40*	*25*	*35*
Providing a visible policing presence		*24*	*71*	*6*
Available to people who wish to speak to an officer or staff member		*29*	*42*	*30*
Weighted data				

A striking feature of the univariate analysis is how many respondents answered ‘don’t know’: depending on the item this figure ranged from 15% to 35%. While just two answered ‘don’t know’ to all 18 items, only 43% gave a definite response to all 18; 11% replied ‘don’t know’ to one, 8% to two, and 5% to three items. Some 13% gave this response to 10 or more items. One interpretation of this divergent spread of responses is that many respondents were unwilling to give a response to an item when they felt they lacked knowledge of the issue concerned. While ‘don’t know’ responses are usually treated as a nuisance in survey analysis, given the way the items were derived they are arguably more meaningful in this instance. Responding ‘don’t know’ to questions about whether police meet various criteria – defined by the focus groups as minimum standards – may itself indicate doubt in the trustworthiness of police.

Scores on the overall confidence and legitimacy items were also low (Table 3). For example, just 37% felt their local police were doing a good or excellent job; 46% agreed that *Most police officers stand up for values that are important to people like me* (a measure of normative alignment); while 44% gave a positive response – above 3 on a 0–6 scale – to the question *To what extent is it your moral duty to do what the police tell you, even if you don’t like how they treat you* (a measure of duty to obey). Levels of trust were somewhat higher, however. Some 64% agreed with the statement *I am happy to accept the ability of the police to intervene in people’s lives*, for example. It therefore seems that while they clearly ‘move’ (or perhaps rather ‘clump’) together, there are clearly differences in public opinion on these measures. While confidence is low, and legitimacy under strain, trust in the police is higher.

**Table 3.** Trust, overall confidence and legitimacy.

Percentages				
Trust	Agree or strongly agree	Neither agree nor disagree	Disagree or strongly disagree	Don't know
I am comfortable allowing the police to decide how to deal with problems of crime and disorder	*61*	*23*	14	2
If I was a victim of a violent crime, I would be content to let the police deal with the matter	*75*	*13*	10	2
I am happy to accept the ability of the police to intervene in people’s lives	*64*	*24*	9	3
				
**Normative alignment**	Agree or strongly agree	Neither agree nor disagree	Disagree or strongly disagree	Don't know
Most police officers have the same sense of right and wrong as I do.	*44*	*24*	*17*	*15*
Most police officers stand up for values that are important to people like me.	*47*	*25*	*15*	*14*
I generally support how most police officers interact with the people in my area.	*50*	*27*	*9*	*13*
				
**Duty to obey** **(to what extent is it your moral duty to ...) (high=more)**	More than 3	3 (mid-point)	Less than 3	Don't know
Back the decisions made by police, even when you disagree with them?	*36*	*22*	38	4
Do what the police tell you, even if you don’t understand or agree with the reasons?	*52*	*20*	25	3
Do what the police tell you to do, even if you don’t like how they treat you?	*44*	*23*	31	3
				
**Overall confidence**	Agree or strongly agree	Fair	Poor or very poor	Don't know
How good a job are local police doing	*37*	*45*	*18*	*0*
Weighted data				

### Measurement properties of the MiPoS items

To explore the measurement properties of the MiPoS items, and to address RQ2, we used Confirmatory Factor Analysis in the statistical package Mplus 7.2. All observed indicators were set to ordinal, and we used Full Information Maximum Likelihood (FIML) estimation, such that cases with some missing values were retained in the analysis. This meant that all but two respondents were retained.

Table 4 shows results from a series of models with one, two and three factor solutions. These models explore whether the 18 survey items can best be considered as indicators of a single latent variable – which one might simply label confidence in the police, or perceptions of police trustworthiness – or whether they are best construed as indicators of two or three distinct latent variables, in the latter case representing the Response, Behaviour/Treatment, and Presence/Engagement domains. Results suggest that the three-factor solution (with no cross-loadings) is the best fit. Only in this model do the RMSEA, CFI, TLI and SRMR scores reach acceptable levels (Hu and Bentler 1999) (all standardised factor loadings were >.76, and all item R^2^ were >.60). The next best fitting model is a two-factor solution where Behaviour/Treatment and Presence/Engagement are combined, but here the approximate fit statistics indicate a less good fit, and the Chi^2^ value is significantly larger than in the three-factor model. It seems therefore that variation in response to the MiPoS items can best be explained by three underlying latent constructs that map onto the domains specified by the focus group work.

**Table 4.** MiPoS domains – confirmatory factor analysis.

	Chi^2^	DF	*p*-value	RMSEA	CFI	TLI	SRMR
3 Factors	1516.5	132	<.00005	0.08	0.97	0.97	0.03
2 Factors (Response and B/T combined)	3461.8	134	<.00005	0.13	0.94	0.93	0.07
2 Factors (Response and *P*/E combined)	2545	134	<.00005	0.11	0.95	0.95	0.05
2 Factors (B/T and *P*/E combined)	2459.2	134	<.00005	0.11	0.97	0.95	0.05
1 Factor	4229.5	135	<.00005	0.14	0.92	0.91	0.07

As might be expected, the three latent variables were strongly correlated, with values ranging from .80 to .84 (see Appendix Table). This is at the margins for acceptable levels in terms of discriminant validity (Rönkkö and Cho 2022). Nonetheless, we proceeded with the three-factor solution, keeping the three domains as separate scales: the fit statistics favour the 3-factor model, and this is how the focus groups specified the domains.

We also used CFA to derive and validate scales of trust and legitimacy. Using the items show in Table 3, we specified a three-factor model (trust, normative alignment and duty to obey) with no cross-loadings and FIML estimation. Model fit was adequate (Chi2 = 190.6; DF = 24; *p* < .00005; RMSEA = .07; CFI = .99; TLI = .99; SRMR = .02), with all standardised factor loadings > .78 and all item R2 > .48. We extracted the factor scores for further analysis.

The Appendix Table shows the correlation matrix and descriptive statistics for the various scales used in this study.

### Association between confidence across the MiPoS domains and other attitudes toward police

To address RQ3 we estimated a series of regression models predicting: the single item gauging views on whether police are doing ‘good job’ locally; the scale representing trust in the police; and the two measures of legitimacy. Modelling proceeded in two steps in each case – first, we fitted a model with the control variables listed above, then we fitted a second model that added the three MiPoS domains.

Considering first the measure of overall confidence (Table 5), we find that confidence across the MiPoS domains is a strong predictor of overall confidence (Model 2).^6^ All three scales have independent statistical effects on overall confidence, with, it seems, Presence/Engagement the most important factor. Conditioning on the MiPoS measures, females tend to have greater overall confidence than males, while those in economically precarious situations, who perceived disorder in their local area, and who had recent unsatisfactory self-initiated contact with police tend to have lower overall confidence. Note also the differences between Model 1 and Model 2 in Table 5. Notably, contact with police, both self- and police-initiated, is a consistent predictor of overall confidence in Model 1, but this largely drops out in Model 2, indicating a mediation effect (in line with the idea that satisfaction with police actions predicts confidence and perceptions of police trustworthiness).

**Table 5.** Regression models predicting overall confidence and trust in police.

Models 1 and 2 are ordinal logistic regression models; Models 3 and 4 are linear regression models								
	Overall confidence				Trust			
	Model 1		Model 2		Model 3		Model 4	
	b	se(b)	b	se(b)	b	se(b)	b	se(b)
Age	0	0	0	0	0.01***	0	0.005***	0
Sex (ref: male)								
Female	0.18+	0.1	0.25*	0.11	0	0.04	−0.02	0.03
Ethnic group (ref: White)								
Asian	−0.07	0.2	−0.08	0.23	−0.20**	0.07	−0.21***	0.06
Black	0.09	0.28	−0.09	0.31	−0.12	0.1	−0.1	0.08
Other	−0.25	0.23	−0.33	0.26	−0.13	0.08	−0.14*	0.07
Economic Precarity (high = more)	−0.08**	0.03	−0.11**	0.04	−0.03**	0.01	−0.03**	0.01
Limiting disability (ref: no)								
Yes	−0.09	0.13	0.13	0.15	−0.13**	0.05	−0.09*	0.04
IMD Decile	−0.01	0.02	−0.02	0.02	0.01	0.01	0	0.01
Victim of crime (ref: no)								
Yes	−0.08	0.19	−0.14	0.21	−0.07	0.07	−0.06	0.05
Perceptions of disorder (high = more)	−0.48***	0.1	−0.55***	0.11	−0.06+	0.03	−0.03	0.03
Worry about crime (high = more)	−0.27**	0.09	−0.15	0.1	−0.03	0.03	0.02	0.03
Self-initiated contact with police (ref: none)								
Yes and satisfied	0.48**	0.15	0.23	0.17	0.10+	0.05	0	0.04
Yes and neutral	−0.41+	0.24	−0.11	0.27	−0.07	0.09	0.04	0.07
Yes and dissatisfied	−1.15***	0.21	−0.46+	0.23	−0.22**	0.08	0	0.06
Police-initiated contact (ref: none)								
Yes and satisfied	0.66***	0.15	0.21	0.17	0.21***	0.05	0.05	0.04
Yes and neutral	−0.25	0.26	−0.52+	0.29	−0.17+	0.09	−0.15+	0.08
Yes and dissatisfied	−0.73*	0.29	−0.18	0.32	−0.55***	0.1	−0.34***	0.09
Newspaper readership (ref: other/none)								
Right of centre	0.07	0.12	0.01	0.13	0.08+	0.04	0.05	0.03
Centre/left	−0.36**	0.13	−0.17	0.15	−0.26***	0.05	−0.20***	0.04
Vote at 2019 election (ref: DN vote)								
Conservative	−0.05	0.07	0.06	0.08	−0.02	0.02	0.02	0.02
Labour	−0.18	0.14	0.17	0.16	−0.08	0.05	0	0.04
Other party	−0.26	0.18	−0.19	0.2	0.06	0.06	0.11*	0.05
**MiPoS measures**								
Response (high = greater confidence)			0.58***	0.15			0.08*	0.04
Behaviour/Treatment (high = greater confidence)			0.79***	0.15			0.41***	0.04
Presence/Engagement (high = greater confidence)			1.63***	0.14			0.06	0.03
Constant	.	.	.	.	0	0.1	0.02	0.08
R^2^	.	.	.	.	0.15		0.43	
N	1414		1414		1415		1415	

^+^ *p* < .1, *p < .05, **p < .01, ***p < .001.

Considering trust in the police (Models 3 and 4 in Table 5), a rather different picture emerges. Here, of the MiPoS scale Behaviour/Treatment has the dominant statistical effect on trust. Response has a much weaker, although still significant association, while Presence/Engagement is non-significant. Conditioning on the MiPoS measures, different control variables emerge as significant predictors: older people tend to trust the police more (as did those who voted for ‘other political parties’), while people with Asian and ‘other’ ethnicities, in economically precarious situations, with limiting disabilities, and who read centre/left newspapers tending to trust the police less, as did those with recent neutral and unsatisfactory police-initiated contact. As in the overall confidence models, we find some evidence that the MiPoS scales mediate the statistical effects of past contact with police on trust. For example, the coefficient for satisfactory police-initiated contact is significant in Model 3 but not in Model 4. Finally, addition of the MiPoS scales significantly increases the model R^2^, from .15 in Model 3 to .43 in Model 4.^7^

Turning to the two components of legitimacy, Table 6 shows the results from four models predicting, first, normative alignment with police (Models 5 and 6), and second felt duty to obey (Models 7 and 8). Results again indicate that the scales derived from the MiPoS items are strong correlates of normative alignment, in particular. Model 6 shows all three components have conditional correlations with this aspect of legitimacy, with Behaviour/Treatment exerting the largest statistical effect by some margin. The R^2^ value for this model is high, .57, indicating that a large proportion of the variation in judgements on this index of legitimacy is explained by the variables in the model, with the MiPoS scales playing a major role (Model 5, without the three MiPoS scales, has an R^2^ value of .15). In relation to duty to obey, only Behaviour/Treatment has a unique association with the legitimacy measure. The R^2^ value for this model is also lower, at .20. Much more variation in duty to obey is left unexplained, possibly indicating that people’s sense that they should obey the instructions of police (or not) stems from a wider range of sources.

**Table 6.** Linear regression models predicting police legitimacy.

	Normative alignment				Duty to obey			
	Model 1		Model 2		Model 3		Model 4	
	b	se(b)	b	se(b)	b	se(b)	b	se(b)
Age	0.005***	0	0.004***	0	0.005***	0	0.004***	0
Sex (ref: male)								
Female	0	0.04	−0.02	0.03	0	0.03	−0.02	0.03
Ethnic group (ref: White)								
Asian	−0.15*	0.08	−0.17**	0.05	−0.07	0.06	−0.08	0.06
Black	−0.27*	0.11	−0.23**	0.07	−0.15+	0.09	−0.12	0.08
Other	−0.12	0.09	−0.13*	0.06	−0.13+	0.07	−0.13+	0.07
Economic Precarity (high = more)	−0.03*	0.01	−0.03**	0.01	−0.03*	0.01	−0.02*	0.01
Limiting disability (ref: no)								
Yes	−0.15**	0.05	−0.11**	0.04	−0.14***	0.04	−0.12**	0.04
IMD Decile	0	0.01	0	0.01	−0.01	0.01	−0.01*	0.01
Victim of crime (ref: no)								
Yes	0.06	0.07	0.07	0.05	−0.04	0.06	−0.03	0.06
Perceptions of disorder (high = more)	−0.07+	0.04	−0.02	0.03	−0.03	0.03	−0.01	0.03
Worry about crime (high = more)	−0.06+	0.03	0	0.02	−0.01	0.03	0.02	0.03
Self-initiated contact with police								
Yes and satisfied	0.16**	0.06	0.02	0.04	0.07	0.05	0.01	0.05
Yes and neutral	−0.13	0.09	0.02	0.07	−0.11	0.08	−0.05	0.07
Yes and dissatisfied	−0.35***	0.08	−0.05	0.06	−0.06	0.07	0.06	0.07
Police-initiated contact								
Yes and satisfied	0.19**	0.06	−0.03	0.04	0.09+	0.05	−0.01	0.05
Yes and neutral	−0.19+	0.1	−0.16*	0.07	−0.17*	0.08	−0.15+	0.08
Yes and dissatisfied	−0.52***	0.11	−0.24**	0.08	−0.18+	0.09	−0.05	0.09
Newspaper readership (ref: other/none)								
Right of centre	0.09*	0.05	0.06+	0.03	0.08*	0.04	0.07*	0.04
Centre/left	−0.18***	0.05	−0.11**	0.04	−0.21***	0.04	−0.17***	0.04
Vote at 2019 election (ref: DN vote)								
Conservative	−0.06*	0.03	−0.01	0.02	−0.06**	0.02	−0.04+	0.02
Labour	−0.11+	0.06	0.01	0.04	−0.02	0.05	0.03	0.04
Other party	−0.05	0.07	0.03	0.05	−0.02	0.06	0.01	0.05
**MiPoS measures**								
Response (high = greater confidence)			0.09*	0.04			0.06	0.04
Behaviour/Treatment (high = greater confidence)			0.56***	0.04			0.26***	0.04
Presence/Engagement (high = greater confidence)			0.08*	0.03			−0.01	0.04
Constant	0.04	0.11	0.06	0.08	0.06	0.09	0.08	0.08
R^2^	0.15		0.57		0.09		0.20	
N	1415		1415		1415		1415	

^+^ *p* < .1, *p < .05, **p < .01, ***p < .001.

Considering the other variables shown Table 6, we again find similarities *and* differences in the correlates of the two different measures of legitimacy. Conditioning on the MiPoS measures, older people tend to have higher scores on normative alignment and duty to obey, as did those who read right-wing newspapers, while those in economically precarious positions, with limiting disabilities, and who read centre/left newspapers tend to have lower scores. By contrast, people from ethnic minority groups tend to feel significantly less normatively aligned with police, but the association between ethnic minority status and duty to obey is more tenuous (albeit still tending negative). Similarly, recent contact with police is more consistently associated with normative alignment than with duty to obey, and again we find evidence of mediation effects. Note, for example, that satisfactory and unsatisfactory self-initiated contact are both significant in Model 5, but both lose significance when the MiPoS variables are added in Model 6.

## Discussion

Four research questions framed the analysis presented above. Addressing each in turn, we find, first, that the majority of British residents do not believe that their local police meet minimum standards of behaviour and performance (RQ1). This was particularly marked in relation to the Response and Presence/Engagement domains but, overall, very few people believe police are meeting all the criteria developed in the focus group work. Second, large numbers of people do not feel the police share their values, nor do they feel a strong sense of moral duty to obey police – we thus provide national level evidence that the legitimacy of the police is indeed currently under strain (RQ2). Yet, levels of trust in the police appear to somewhat higher, indicating nuance in the ways people currently think about policing.

Third, scales derived from the MiPoS items proved to be strongly associated with overall confidence, trust, and legitimacy (RQ3). In line with the predictions of procedural justice theory, beliefs about the way officers treat and relate to people and their community seem the most important domain in this regard. Yet, there is again evidence of nuance and variation. Presence/Engagement, for example, was a strong predictor of overall confidence in police.

Turning to RQ4, our findings have implications for efforts to address recent declines in public confidence. Recall, for example, that people had more confidence in Behaviour/Treatment than in Response and Presence/Engagement. There has been a recent emphasis within and around policing on procedural justice training and other relational skills-building and procedures (e.g. the new Policing Code of Ethics published by the College of Policing – CoP 2024), and on recruiting the right people for the job while ‘weeding’ out those already in post who are unsuitable (and at the extreme criminal in their behaviour).^8^ These are vital issues for the police service to address. However, they are being enacted in the context of growing crime and non-crime demand on the police (HMICFRS 2022) and continued fall-out from ‘austerity’ policies in the 2010s. These have stretched police response capacity whilst triggering often swingeing cuts to neighbourhood policing. These pressures seem to have damaged public views on police Response and Presence/Engagement, with the latter particularly negative – and strongly associated with overall confidence in police.

It seems therefore that efforts to improve police-community relations need to look beyond the behaviour of individual officers to encompass organisational activity and responses. A wider set of organisational priorities and resource allocation decisions, and economic stresses and strains, shape public attitudes towards police. Addressing the current ‘crisis’ thus requires more than improvements to officer behaviour. It may be telling in this regard that economic precarity was associated with lower levels of confidence. People experiencing economic stress may rely on police more, since they are more likely to live in higher crime areas (TASERD 2023), have fewer resources to deal with problems in other ways, and thus be more inclined to feel let down when police seem absent or unengaged (they may also be treated differently by officers, of course). Better resourcing of neighbourhood policing, placing more police officers in communities to engage, build relationships and provide visible oversight may be just as important as improving the way individual officers behave (Kimaram *et al.* 2023).

Another consequence of inadequate neighbourhood police presence may be formation of opinions based on blue light responses and media portrayals (Intravia *et al.* 2020). Recent high-profile cases involving corruption, malpractice and mistreatment seem likely to have contributed to declines in public confidence, and indeed legitimacy (EVAW 2021). Positive police-public interactions rooted in everyday engagement offer opportunities to address this. As in other studies (Bolger *et al.* 2021), we found a strong correlation between encounters with officers and attitudes towards police, reinforcing the idea that investment in neighbourhood police presence might enhance public attitudes by demonstrating that police are available, representative, and ready to interact, listen, and respond to community concerns. On this basis, the Labour government’s promise to ‘put communities back at the heart of policing’ and its commitment to introduce a new Neighbourhood Policing Guarantee^9^ appear important, if as yet unachieved and challenging, policy aims.

Most of the discussion above has been framed in terms of confidence in police, both in terms of the MiPoS and current concerns about police-public relations. Yet, our survey also comprises one of the few nationally representative studies to include measures of trust and legitimacy. Here, we find that frequent conflation of confidence and legitimacy in public discourse is not unjustified. Levels of confidence are low, significant numbers of people do not believe police share their moral values, and our data seem to support the idea of a legitimacy crisis. Views on Behaviour/Treatment have the strongest correlation with legitimacy, reinforcing the idea that a focus on procedural justice is central to efforts aimed at redressing this deficit. But other components of confidence also correlated with the normative alignment component of legitimacy, indicating that there may be other, more organisationally-inclined, ways to do so. To put it another way, police should not just rely on officers ‘doing policing better’ to address challenges to legitimacy; they also need to think about the response to crimes and other events, and whether people feel police are engaged and present in their area.

Public *trust* in the police appears somewhat different. Not only did the correlates of trust differ from those of, particularly, overall confidence, but levels of trust were significantly higher. Why this might be the case is currently rather unclear, although it is worth noting that Behaviour/Treatment appeared most important for trust, and this was the MiPoS domain with the most positive scores. The trust items (e.g. ‘*If I was a victim of a violent crime, I would be content to let the police deal with the matter’)* may also wrap up a sense of reliance on police in the absence of other options (who else is going to deal with violent crime) and the ‘inevitability’ of police (there needs to be an institution that interferes in peoples’ lives to deal with crime and disorder). Future empirical work could profitably explore in more detail the potential role of such views, related not to the performance of the police or even the extent of crime and disorder but to what they *represent*, in informing trust and other attitudes.

Finally, we return to the distinction between ‘instrumental’ and ‘relational’ drivers of confidence, trust and legitimacy. Our analysis of the MiPoS survey items indicated that not only did they load onto the domains (i.e. the latent variables) agreed by the focus groups, but these domains were themselves highly intercorrelated. It seems that people find it hard to think about the ‘what’ of policing without also thinking about the ‘how’. These are not separate issues but are intimately bound together. Further research might profitably explore the ways in which people construe these apparently different aspects of police behaviour, and consider the extent to which the distinction commonly drawn between them is justified. That said, it is worth reiterating that our findings are broadly in line with the idea that affective and relational concerns about fairness are the most important aspects of peoples’ judgements of police, and seem to drive, in particular, trust in and the legitimacy of police.

### Limitations

A major limitation to this paper, and the survey work using the MiPoS, is the level of ‘don’t know’ responses to the specified items. While we argued that this is itself indicative of a certain lack of confidence, it clearly would be better to avoid such a large proportion of ambiguous responses. Future efforts using the items and ideas included in the MiPoS could consider different question formulations: one idea might be to frame the items with the prefix ‘How much confidence do you have that … ’, with responses on an appropriate scale (‘A great deal of confidence’ … ‘no confidence at all’), and/or to avoid providing a ‘don’t know’ response category to respondents. More broadly, if some or all of the MiPoS items are to be included in future work, further item testing and investigation of their reliability and validity will be needed.

Inevitably, we cannot trace the causal pathways implied by current theory and the regression models presented above; most obviously, that confidence in the police precedes trust and legitimacy judgements. While it seems likely that assessments of the success of police across the areas covered by the MiPoS domains form the basis of trust and legitimacy – comprising, for example, the evaluations that in classical trust theory inform the expectations which underpin trust – it is equally plausible to suggest that overall judgements of police legitimacy provide a resource or heuristic for making assessments of specific police behaviours. More longitudinal and experimental work is needed to explore these questions, the answers to which may provide important insight into policies aiming to address currently low levels of trust, confidence and legitimacy.

## Conclusion

In this paper we have outlined and investigated a set of survey items derived via an iterative focus group methodology aimed at developing a minimum standard of police service delivery. Taken together, our findings suggest that the MiPoS items constitute a useful set of questions with which to probe people’s views of local policing. While they do not on an individual basis depart radically from the kind of survey items currently used to gauge public attitudes towards police, they add considerable nuance and, collectively, they seem to capture much of what is important to people when they are thinking about whether they trust, and judge legitimate, the police.

The widespread perception that police are failing to meet appropriate standards of service delivery, the strong correlations between these judgements and overall confidence, trust and legitimacy, and the low levels of confidence and legitimacy we identify all suggests that talk of a legitimacy crisis in British policing may not be hyperbolic. Certainly, large sections of the population now seem to hold decidedly negative views of the police. It would seem that a focus on procedural justice, which has become something of a go-to answer to such challenges, may be necessary but insufficient to address this crisis. A wider response that takes in the way the police respond to public calls for assistance and, perhaps particularly, increases the level of police visibility in and engagement with communities, is required. The new Labour government, and others in policing, seem to be making moves in this direction. But whether such a reconfiguration is feasible given resource constraints and other pressures on police remains to be seen.

## Notes

1 https://www.lboro.ac.uk/research/crsp/minimum-income-standard/.

2 Address Based Online Surveying (ABOS) is a survey methodology that allows online surveying of a random sample of the general public instead relying on a panel of people who select themselves into the study.

3 A scale derived from CFA in Mplus, measured by seven items probing ‘how much of a problem’ respondents thought behaviours such as littering, vandalism and speeding cars were in their local area.

4 A scale derived from CFA in Mplus, measured by seven items asking respondents ‘how worried’ they were about falling victim to crimes like burglary/theft by housebreaking, mugging/robbery, and fraud.

5 The Index of Multiple Deprivation (IMD) is a measure of neighbourhood deprivation (Noble *et al.* 2019). Lower Layer Super Output Area (LSOA) is a census-based small area classification (in England and Wales LSOAs have a resident population ranging from 1,000 to 3,000 people).

6 Because the ‘good job’ measure is on a 5-point ordinal scale we used ordinal logistic regression. A Brant test of the proportional odds assumption could not be calculated because not all independent variables could be retained in the binary logit models used to calculate the test. However, multinomial logistic regression and linear regression models with the same covariates gave very similar results, so we proceeded with the ordinal model as a compromise between fidelity to the nature of the response variable and ease of presentation.

7 We considered potential issues of multi-collinearity and heteroskedasticity in the linear regression models. The highest Variance Inflation Factor for any explanatory variable in any model was 4.34 (for the Behaviour/Treatment scale), which does not indicate excessive collinearity (O’Brien 2007). Breusch–Pagan/Cook–Weisberg tests for the models did not suggest any problems with heteroskedasticity.

8 One example is the Metropolitan Police’s Op Onyx programme, which is reviewing the cases of officers previously investigated but not dismissed for sexual offences or domestic abuse cases with the aim of ensuring they are removed from service if appropriate.

9 https://www.gov.uk/government/news/more-bobbies-on-the-beat-as-pm-puts-peoples-priorities-first.

## Appendix table

Scales used in analysis: descriptive statistics and correlation matrix

**Table:**

	Mean	SD	1	2	3	4	5	6	7	8	9	10
Response (1)	−0.03	0.78	1									
Behaviour/Treatment (2)	−0.04	0.78	0.84	1								
Presence/Engagement (3)	−0.03	0.83	0.80	0.82	1							
Overall confidence (4)	3.16	0.88	0.67	0.68	0.71	1						
Trust (5)	−0.01	0.73	0.54	0.60	0.50	0.52	1					
Normative alignment (6)	−0.02	0.78	0.64	0.72	0.62	0.63	0.79	1				
Duty to obey (7)	−0.01	0.64	0.34	0.39	0.30	0.35	0.57	0.56	1			
Perceptions of disorder (8)	0.00	0.69	−0.10	−0.10	−0.10	−0.20	−0.10	−0.10	−0.10	1		
Worry about crime (9)	0.01	0.76	−0.20	−0.10	−0.10	−0.10	−0.10	−0.20	−0.10	0.60	1	
Economic precarity (10)	5.59	1.83	−0.10	−0.10	−0	−0.10	−0.10	−0.10	−0.10	0.29	0.33	1
